# Facilitators and barriers to the consumption of traditional foods among adults in Zimbabwe

**DOI:** 10.1186/s42779-022-00121-y

**Published:** 2022-02-19

**Authors:** Prosper Chopera, Praise R. Zimunya, Felistas M. Mugariri, Tonderayi M. Matsungo

**Affiliations:** Department of Nutrition, Dietetics and Food Sciences, University of Zimbabwe, Mt Pleasant, PO Box MP 167, Harare, Zimbabwe

**Keywords:** Traditional food, Indigenous, Facilitators, Barriers, Zimbabwe

## Abstract

**Background:**

Traditional foods have superior nutritional composition; however, they are largely absent from the Zimbabwean diet.

**Objective:**

To identify barriers and facilitators to the consumption of traditional foods among adults aged 18–69 years in Zimbabwe.

**Methods:**

An online-based cross-sectional survey was conducted among adults aged 18–69 years in Zimbabwe. The questionnaire was based on a socio-ecological model designed to assess barriers and facilitators at the individual, interpersonal, community, and national levels. Data were analysed using Microsoft Excel and SPSS version 20 software package. The level of significance was set at p < 0.05. Ethical approval was obtained from the Medical Research Council of Zimbabwe (MRCZ/B/1931).

**Results:**

The study enrolled 440 participants. Traditional food consumption was low in this population with only 9.3% consuming these foods daily. At the individual level, 44.4% of study participants mentioned their consumption of traditional foods is facilitated by generational factors, while the most important barrier at this level was the inconvenience in accessing and preparing traditional foods (33.2%). At the community and national levels, the most important facilitator was family members (26.2%) and lack of environmental contaminants (38.9%), respectively, while most important barrier at the community and national levels was their residential location or residence (31.8%) and aggressive marketing of processed foods (47.8%), respectively.

**Conclusions:**

Consumption of traditional foods was low in general. Generational factors, family contribution, and food safety impact the consumption of traditional foods among adults in Zimbabwe. The food environment, particularly commercial advertising of alternative foods, is a deterrent. Therefore, interventions to promote the consumption of traditional foods must take into account these factors at every stage of the socio-ecological model.

## Introduction

Traditional and indigenous foods, by definition, include all wholesome foods that have social and cultural value and preference, are accessible, prepared using local natural ingredients, and are specific to a region or country [1]. However, the concept of traditional food is very dynamic, complex and variable, still four dimensions to their definition have been established: time, place, know-how, and cultural meaning [2]. These cultural foods are passed on from one generation to another [3] and were common in societies before the modernisation and industrialisation *“westernisation”* [4]. There is a general surge in the interest in traditional foods due to the increased awareness of the relationship between diet and non-communicable diseases (NCDs) [5] and communicable diseases like COVID-19 [6, 7].

The traditional African diet is mainly plant-based consisting of small grains (millet and sorghum), dark green leafy vegetables, wild fruits, starchy stems, and root tubers. Though largely cereal-based, a few animal source foods were added to the diet such as fish and game meat [8]. In Zimbabwe, traditional foods consist of small grains eaten with a variety of boiled or fried wild vegetables, seeds, and or game meat (Table 1). The nutrition transition is characterised by increased consumption of processed and energy-dense foods coupled with sedentary lifestyles [9]. In Zimbabwe, there is now an increase in the consumption of wheat, rice and maize products at the expense of traditional staple cereals, roots, and tubers. Indigenous fruits and vegetables have been largely replaced by exotic fruits and vegetables in the common household [10].

**Table 1:**
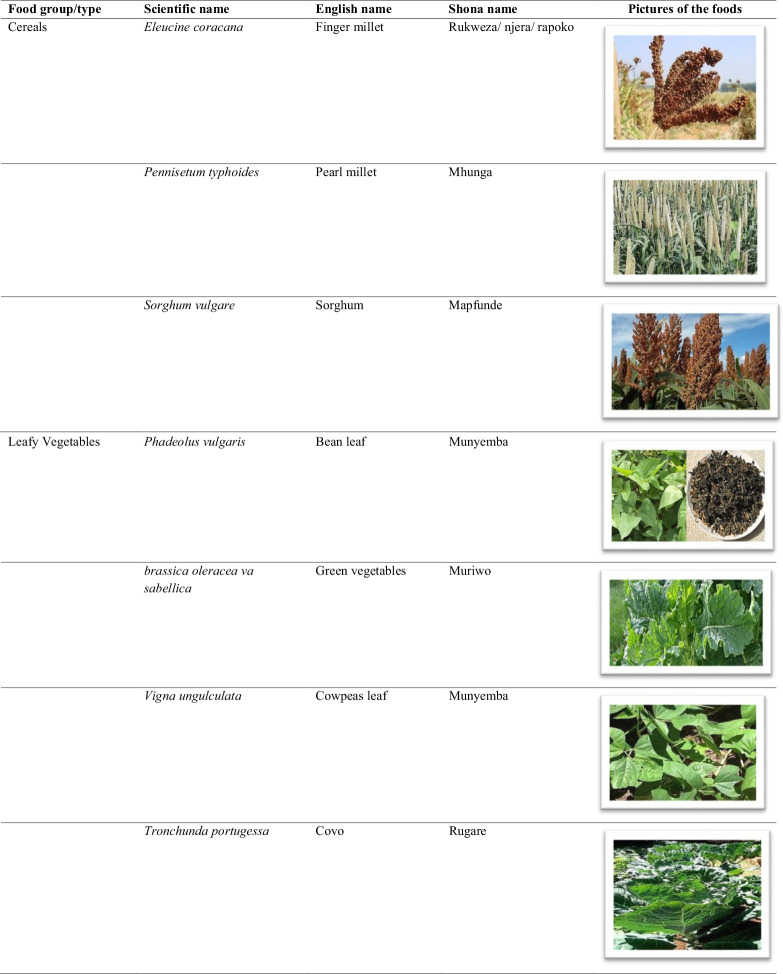

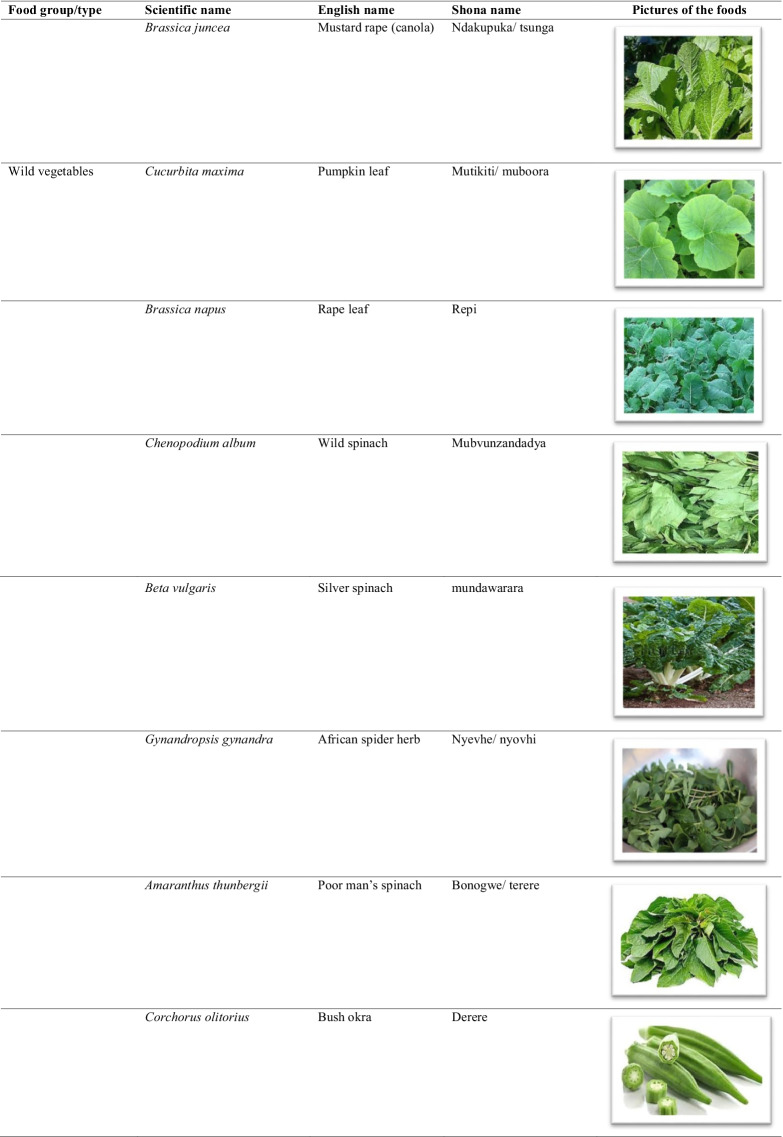

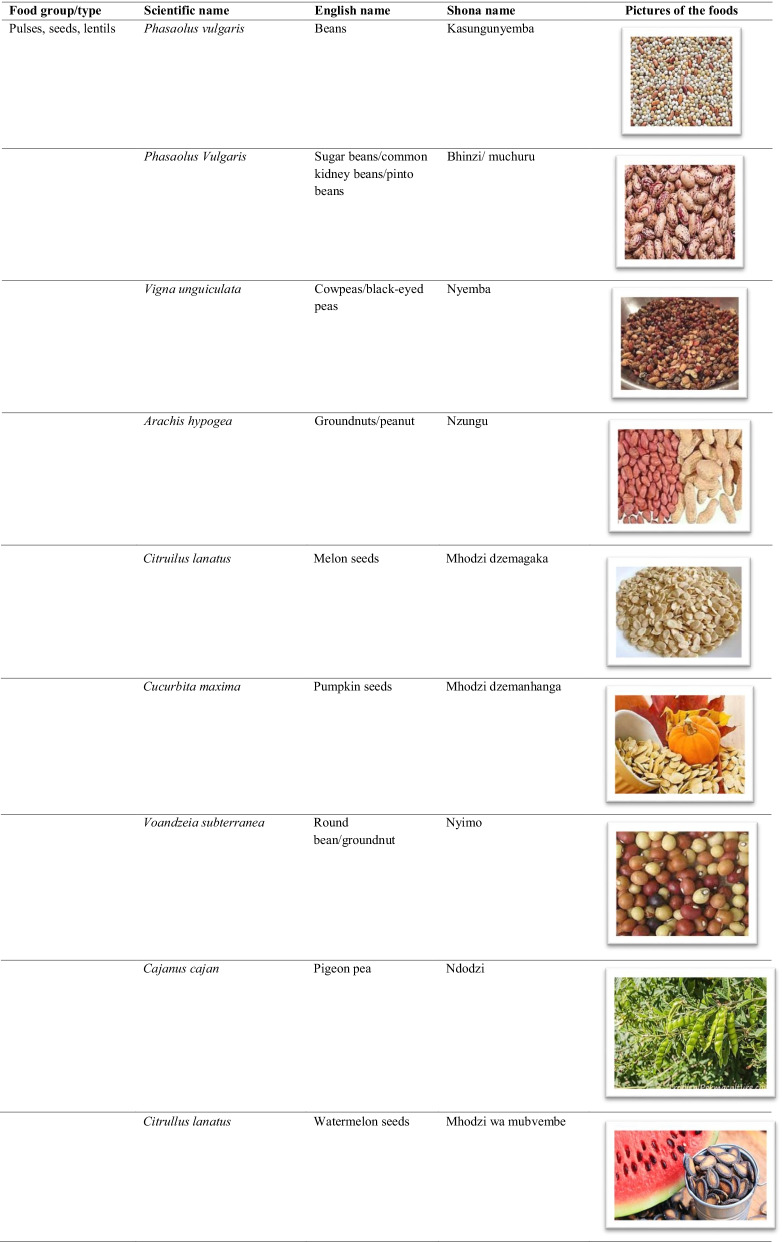
List of some common traditional foods in Zimbabwe

The food names were hugely informed by Gomez [32], the food pictures were obtained from www.google.com

Most traditional foods have been shown to contain healthy components such as antioxidants [11, 12]. In addition, the traditional and indigenous fruits and vegetables are usually drought resistant [13]. Subsisting mainly on traditional diets, therefore, has the potential to solve two problems, i.e.: (1) rising food insecurity due to climate change effects (2) increase in non-communicable diseases (NCDs).

Dietary changes over time can be influenced by many factors such as geographical, environmental, social, and economic factors [14]. This may mean that complex interactions such as migration, income, prices, individual preferences, beliefs, and cultural traditions influence food choices continuously as they don’t remain static over time. Nutrition transition, as a global event, has caused changes in the quality and quantity of food consumption patterns in many countries, races, social classes, and cultures. Zimbabwe is no exception. There is evidence that nutrition transition comes with a rise in the prevalence of diet-related NCDs such as obesity and type 2 diabetes mellitus [15].

Zimbabwe is a country experiencing rapid urbanisation. Furthermore, the country has seen an increase in trends of diseases such as type 2 diabetes, cancer, and metabolic syndrome [16]. Zimbabwe’s Human Development Index score in 2019 stood at 0.571 ranking it 150 out of 189 countries [17], while the Gender Inequality Index score of Zimbabwe was 0.527, ranked it 129 out of 162 countries in 2019 [17]. Since the year 2000, the country has also registered a decline in some socio-economic indicators mainly attributed to economic recession, political challenges, and persistent climate-induced humanitarian crises [18] This is a huge threat to household food security and slows progress on the attainment of the Sustainable Development Goals (SDGs) by 2030.

Current climate adaptation strategies include animal and crop diversification with emphasis on drought-tolerant crops such as sorghum and millet [13]. These crops that have largely been replaced by exotic crops were part of African traditional diets for ages. Available evidence has shown that the reception or rejection of food is a complex phenomenon, which is ever-changing and variable. Furthermore, this multi-dimensional system is influenced by an individual’s attitude and by individuals interacting within a community in different contexts and over different periods [19]. Studies conducted in other countries have revealed that personal factors that influence eating patterns can include attitudes, beliefs, food preferences, self-efficacy, and biological changes, while environmental factors can include the immediate social environment such as family, friends, and peer networks, and other factors such as school, fast food outlets, street vendors, and social and cultural norms [20, 21]. There is a dearth of information concerning patterns and reasons for traditional food consumption in Zimbabwe [22]. The perceptions and attitudes around traditional food consumption in other countries like South Africa are well documented and reveal that these foods have been regarded as poor man’s food hence the decreased popularity [23]. The few studies conducted in Zimbabwe have looked at reasons for growing them and not consuming them. Traditional foods have been cultivated for subsistence purposes and to build resilience in local farming communities [24, 25] as well as in other African countries. They also serve commercial purposes albeit to a lesser extent [26]. Considering the rich biodiversity in Zimbabwe, communities should be encouraged to consume indigenous and traditional foods as part of the dietary diversification strategy for sustainable nutrition and health.

Therefore, this study was designed to assess the patterns, facilitators, and barriers to consumption of traditional and/or indigenous foods in Zimbabwe utilising a socio-ecological approach. The social-ecological approach has been used to explore physical activity interventions [27], promote healthy eating in schools [28] and to understand barriers and facilitators to consumption of traditional foods by Gaudin et al. [29, 30], and Roudsari et al. [31]. The socio-ecological approach when applied helps to clarify factors affecting traditional food consumption at each level of society and considers the complex interplay between individual, interpersonal, community and environmental factors.

## Methods

### Study design and setting

**Fig. 1:**
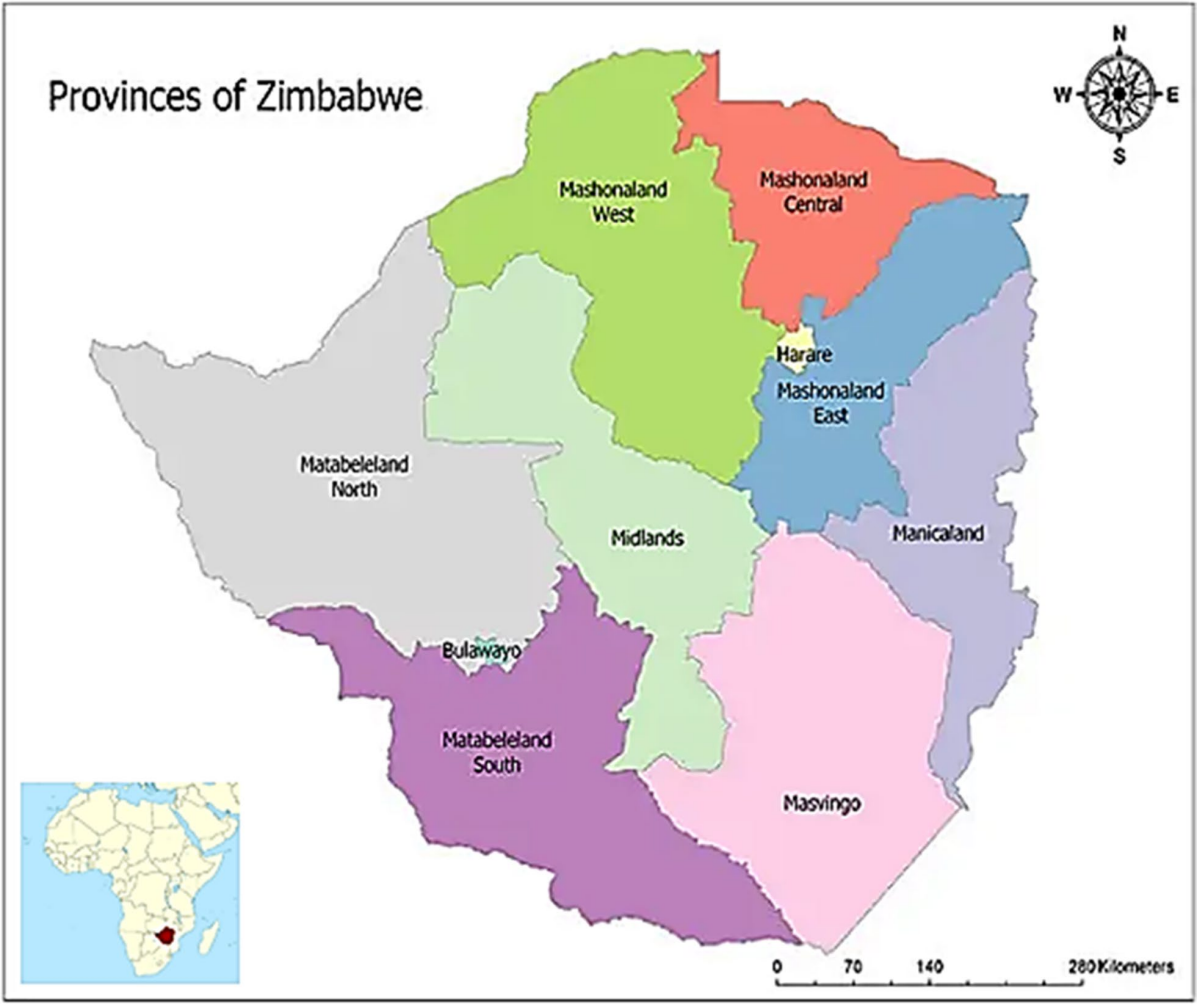
Map of Zimbabwe with provinces that took part in the survey. *Source*: https://www.mappr.co/political-maps/zimbabwe/

The cross-sectional study utilized an online poll approach to collect quantitative data from adults 18–69 years in Zimbabwe. The Southern African country is landlocked with 10 provinces and 61 administrative districts (Fig. 1). The population of Zimbabwe is 13 061 239 million according to the 2012 census (ZIMSTAT) with about 40% below the age of 45 years old and a proportion of males and females of 48 and 52 per cent, respectively [33]. According to a 2012 Census report, 99.6% of the population is of African origin. Although Zimbabwe has 16 official languages, Shona and Ndebele are the main native languages, while English is the formal language widely used in administration, law, and schools. The literacy rate is 94% [33].

### Data collection and tools

A questionnaire assessing the barriers and facilitators to consumption of traditional foods was administered using a web-based survey hosted on the Survey monkey platform (SurveyMonkey, California, USA). The survey language was English. The questions were based on two ecological models developed by Gaudin et al. [29]. One model in a series of concentric circles proposes possible facilitators at the individual, interpersonal, community, and environment/ national levels. The second model proposes possible barriers at the same levels. The questionnaire was pre-tested in a small group of adults before it was circulated in the following manner; a department mailing list was obtained and WhatsApp contacts of the same individuals. The individuals were informed that this was a pretest. Feedback was solicited on clarity of questions, order of questions, skip patterns, timing, and task difficulty. Questions were adapted accordingly and the questionnaire was finalised. The survey tool was distributed to participants via known institutional mailing lists through partner organizations, and social media platforms mainly WhatsApp, Facebook, and Twitter. Only one response was allowed per device hence increasing the reliability of the data. The questionnaire took approximately 6 min to complete.

### Body Mass Index (BMI) and Silhouette matching test

A nine figural BMI-SMT (Body Mass Index-Silhouette Matching Test) was used to collect information on perceived anthropometry [34]. The participants were asked to choose one gender-based silhouette that they thought best described their body size with 1 being thinnest and 9 being the heaviest.

### Participants and sampling

All adults living in Zimbabwe aged between 18 and 69 years were eligible for the study. The sample size was calculated using the Cochran formula using α = 0.05 and 95% significance level and 50% prevalence of traditional foods consumption. A sample size of 384 was determined. After factoring in the non-responders’ rate of 10%, the final sample size was adjusted to 427.

### Ethics

Ethical approval was obtained from the Medical Research Council in Zimbabwe (MRCZ) (MRCZ/B/1931). Electronic consent was sought from study participants through a consent question at the beginning of the online questionnaire which asked participants if they consented to participate in the survey.

### Data analysis

Data were exported to SPSS version 20 (SPSS Inc. Chicago, IL), coded, and cleaned. The Shapiro–Wilk test was used to test for the normality of data. Continuous variables with a normal distribution were presented as means and standard deviation, while categorical data were presented as frequencies and percentages. The Chi-square test was used to explore associations between categorical variables and Fisher’s exact test when cell count was less than 5. The level of significance was set at p < 0.05.

## Results

### Characteristics of participants

The web-based survey was stopped in July 2020. A total of 440 participants took part in the survey, the majority were females (69.3%) and 30.7% were male. Most participants (94.6%) had attended university or tertiary college and 95.6% were Christians. The majority of the participants (47.7%) were aged between 18 and 30 years with the least representation from the age group 50–69 years (4.5%). The participants’ age ranged from 18 to 69 years (M = 32.2, SD = 9.3). Most of the participants (53.8%) were from the capital city Harare (Table 2).

**Table 2:** Sociodemographic characteristics of the participants

Variable	n	%
*Gender*		
Male	126	30.7
Female	284	69.3
*Age category (years)*		
18–30	190	47.6
31–40	144	36.1
41–49	47	11.8
50–69	18	4.5
*Education level*		
Ordinary level	6	1.5
Advanced level	16	3.9
Tertiary level	388	94.6
*Employment status*		
Employed	282	68.9
Not employed	127	31.1
*Residence*		
Urban province	326	62.8
Rural province	98	37.2
*Religion*		
None	4	1
Christian	389	95.6
Muslim	1	0.2
ATR	4	1.0
Apostolic sect	4	1.0
Other	5	1.2

*ATR* African Traditional Religion

### Traditional food consumption and sociodemographic characteristics

Traditional food consumption was low in this population with only 9.3% consuming these foods daily and 20% never having consumed traditional foods (Table 3). A large proportion of females (71.1%) consumed traditional foods daily compared to 28.9% of their male counterparts. There was however no significant difference in frequency of consumption across gender (p = 0.05), age group (p = 0.89), education level (p = 0.36), residential status (p = 0.06), who the respondent lived with (p = 0.91), nutritional status (p = 0.83), religion (p = 0.71) and marital status (p = 0.32). There was a significant difference in frequency of consumption and employment status (p = 0.004) with higher consumption in the employed group.

**Table 3:** Traditional food consumption trends and selected participant characteristics

	Traditional food consumption frequency n (%)						
	Never	Once per month	1–2 times per week	> 2 times per week	> 3 times per week	Daily	*P* value^1^
Total	20 (4.9)	123 (30.1)	96 (23.5)	64 (15.6)	68 (16.6)	38 (9.3)	
*Gender*							
Male	4 (20)	41 (33.3)	23 (24)	29 (45.3)	17 (25.4)	11 (28.9)	0.05
female	16 (80)	82 (66.7)	73 (76)	35 (54.7)	50 (74.6)	27 (71.1)	
*Age category in years*							
18–30	10 (52.6)	54 (44.3)	45 (46.9)	32 (51.6)	29 (46)	19 (54.3)	0.89
31–40	7 (36.8)	50 (41)	38 (39.6)	17 (27.4)	24 (38.1)	8 (22.9)	
41–49	1 (5.3)	13 (10.7)	9 (9.4)	9 (14.5)	8 (12.7)	6 (17.1)	
50–69	1 (5.3	5 (4.1)	4 (4.2)	4 (6.5)	2 (3.2)	2 (5.7)	
*Education level*							
Ordinary level	0 (0.0)	0 (0.0)	2 (2.1)	2 (3.1)	1 (1.5)	1(2.6)	0.36
Advanced level	1 (5.0)	3 (2.4)	6 (6.3)	3 (4.7)	3 (4.5)	0 (0.0)	
Tertiary level	19 (95.0)	120 (97.6)	88 (91.7)	88 (91.7)	63 (94.0)	37 (97.4)	
*Employment status*							
Employed	4 (70)	101 (82.1)	61 (63.5)	41 (65.1)	43 (64.2)	20 (52.6)	0.004*
Not employed	6 (30)	22 (17.9)	35 (36.5)	22 (34.9)	24 (35.8)	18 (47.4)	
*Province*							
Urban	10 (55.6)	78 (70.9)	52 (58.4)	27 (46.6)	37 (60.7)	23 (65.7)	0.06
Rural	8 (44.4)	32 (29.1)	37 (41.6)	31 (53.4)	24 (39.3)	12 (34.3	
*Who do you stay with*							
Family	17 (85.0)	105 (85.4)	83 (86.5)	60 (93.8)	61 (89.7)	34 (89.5)	0.91
Friends	0 (0.0)	1 (0.8)	1 (1.0)	0 (0.0)	0 (0.0)	0 (0.0)	
Alone	3 (15.0)	17 (13.8)	12 (12.5)	4 (6.3)	4 (10.5)	4 (10.5)	
*Nutritional status*							
Underweight	2 (10)	5 (4.1)	3 (3.1)	3 (4.7)	3 (4.4)	0 (0)	0.83
Normal	9 (45)	42 (34.1)	35 (36.5)	26 (40.6)	24 (35.3)	13 (34.2)	
Overweight	5(25)	56 (45.5)	45 (46.9)	27 (42.2)	35 (51.5)	19 (50)	
Obesity	4 (20)	20 (16.3)	13 (13.5)	8 (12.5)	6 (8.8)	6 (15.8)	
*Religion*							
None	1 (5.0)	1 (0.8)	1 (1.1)	0 (0.0)	1 (1.5)	0 (0.0)	0.72
Christian	18 (90.0)	117 (95.9)	92 (96.8)	60 (93.8)	63 (95.5)	37 (97.4)	
Muslim	0 (0.0)	0 (0.0)	0 (0.0)	1 (1.6)	0 (0.0)	0 (0.0)	
AFR	0 (0.0)	2 (1.6)	0 (0.0)	0 (0.0)	1 (1.5)	1 (2.6)	
Apostolic sect	0 (0.0)	1 (0.8)	1 (1.1)	2 (3.1)	0 (0.0)	0 (0.0)	
Other	1 (5.0)	1 (0.8)	1 (1.1)	1 (1.6)	1 (1.5)	0 (0.0)	
*Marital status*							
Single	10 (50.0)	52 (42.3)	45 (47.4)	31 (48.4)	27 (40.3)	25 (65.8)	0.32
Married	10 (50.0)	68 (55.3)	46 (48.4)	31 (48.4)	36 (53.7)	11 (28.9)	
Divorced	0 (0.0)	1 (0.8)	3 (3.2)	2 (3.1)	2 (3.0)	0 (0.0)	
Widowed	0 (0.0)	2 (1.6)	1 (1.1)	0 (0.0)	2 (3.0)	2 (5.3)	

*ATR* African Traditional Religion

^1^*p* value based on Pearson’s Chi-square test and Fisher’s exact test for cell counts < 5

*Significant at *p* < 0.05

### Facilitators of traditional food consumption

The most common factor that facilitated traditional food consumption at the individual level was a cross-generational influence (44.4%) (Table 4). This meant that these foods had been successfully handed down generations in about a third of the respondents and the presence of older generations in families was facilitating consumption. The most common facilitator at the interpersonal level was family (42.5%) meaning traditional foods were part of the family diet due to influence from other members such as parents and siblings. At the community level, the most common facilitator was the influence of family and elders in the community (26.2%) meaning the extended community was playing a role in influencing individuals by ensuring these foods are present at community and other gatherings. At the national/environmental level, 38.9% selected the reason that the assurance of the absence of contaminants such as mercury in traditional foods would encourage them to eat. Most traditional foods are not processed commercially but through artisanal methods that are largely unregulated. The government and environmental bodies have a role to play in ensuring that these foods are safe.

**Table 4:** Facilitators and Barriers to traditional food consumption

Variable	n	%
*Facilitators to traditional food consumption*		
Individual level		
Generational	165	44.4
Lifestyle	88	23.7
Convenience	46	12.4
Preparation skills	19	5.1
Cost	28	7.5
Other	26	7.0
Interpersonal level		
Family influence	149	42.5
Peer influence	18	5.1
Social norms	45	12.8
Values	130	37.0
Other	9	2.6
Community level		
Family/elder contribution	87	26.2
Social norms	42	12.7
Community values	82	24.7
Residential location	50	15.1
Local business	51	15.4
Other	20	6.0
Environment level		
Government regulations	67	20.9
Sustainability	55	17.7
Adequate marketing	43	13.8
Environmental contaminants	121	38.9
Other	27	8.6
*Barriers to traditional food consumption*		
Individual level		
Generational	36	9.8
Lifestyle and preferences	63	17.1
Convenience	122	33.2
Skills of preparation	44	12.0
Cost	82	22.3
Other	21	5.7
Interpersonal level		
Lack of family influence	48	14.0
Lack of peer influence	37	10.8
Not a social norm	152	44.2
Interpersonal values	66	19.2
Other	41	11.9
Community level		
Lack of family support	71	21.7
Not a social norm	53	16.2
Community values	19	5.8
Residential location	104	31.8
Local businesses not in support	68	20.8
Other	12	3.7
Environment level		
Absence of government laws and regulations Protecting traditional foods	84	26.9
Sustainability not guaranteed	47	15.1
Environmental contaminants	13	4.2
Aggressive marketing	149	47.8
Absence of pro-traditional foods regional bodies	17	5.4
Other	2	0.6

### Barriers to traditional food consumption

The lack of convenience in the preparation of traditional foods resulted in it being the most common barrier to traditional food consumption at the individual level (33.2%). Proper preparation requires knowledge and skills that are normally passed on from older generations. Furthermore, most preparation methods are lengthy and cumbersome. Small grain products for example are not highly prevalent in supermarkets for a variety of technical reasons that include extreme levels of the drudgery associated with the current small grain production systems. This also affects availability at the household level. At the interpersonal level, it was noted that traditional food consumption ‘not being a social norm’ was a barrier by 44.2% of the participants. Meaning that eating traditional foods was not the standard in and across families. At the community level, the residential location was cited by 31.8% of the respondents as a barrier. This meant that for individuals residing in communities that have neither access to such foods in the shops nor land to cultivate these foods, frequent consumption was difficult. At the environmental level, 47.8% of participants responded to the aggressive marketing of other foods as a major barrier to traditional food consumption. The increased visibility of for example fast foods meant traditional foods are largely ignored in most shopping baskets. This could be indicating the impact of evolving food systems, particularly in urban areas.

## Discussion

Traditional food consumption has gradually decreased over the years despite their health benefits. Therefore, this study set out to investigate the facilitators and barriers to traditional food consumption among adults aged 18–69 years in Zimbabwe. In this study, traditional food consumption was low in this population. Contrary to our expectations being employed was positively associated with the consumption of traditional foods. For example, due to their busy work schedules, due to “time scarcity,” we would have expected low consumption among the working class [35]. Traditional dishes such as those made from offals or cow hooves take hours to cook to a softened texture. The processing of millet grains from thrashing to winnowing to pounding to make a thick staple called *sadza* is time-consuming and laborious. If not done properly, the final product will end up with sand grains thus unpleasant to eat. A study in Canada reported the contrary that employment acts as both a facilitator and a barrier to traditional food consumption, rendering the effect undetectable [30]. In Northern Quebec, Canada employment status was a facilitator to consumption of traditional foods probably due to access to disposable income [29]. However, on the contrary, parental employment and work-life stress are normally associated with a less healthy family food environment [36]. Previous studies have reported that urban dwellers are less likely to consume indigenous foods as compared to their counterparts in rural or peri-urban areas. So, the current finding is interesting and encouraging within the premises of promoting consumption of traditional foods in Zimbabwe’s urban areas where consumption of traditional foods tends to be driven by affordability. Promoting traditional food consumption through nutrition education “demand creation” should be coupled with measures in the agriculture sector to conserve and/or cultivate the traditional species. The government of Zimbabwe may initiate the acquisition and management of germplasm of indigenous and traditional varieties [37]. Therefore, multisectoral and collaborative research is warranted for the conservation of the traditional varieties owing to their nutritional benefits.

### Facilitators of traditional food consumption

We found that the strongest facilitators were (1) cross-generational influence, (2) family support system, and (3) food safety guarantees. A study in Cree in Canada also reported that individuals aged above 40 years were likely to consume traditional foods [30] This highlights the importance of cross-generational influence as a facilitating factor. In Zimbabwe, the old generation places more importance on traditional foods as compared to the younger generation since the old generation grew up in rural areas and with an abundance of wild fruit and vegetables. The young generation is, therefore, less likely to consume nutritious indigenous foods due to different exposures. These same findings were also reported in Northern Quebec in Canada that traditional knowledge was a key factor influencing traditional food consumption in a Cree community [29]. Therefore, the current findings are important as they reveal that social behaviour change interventions aimed at promoting consumption of traditional foods should be built on pre-existing knowledge and practices, which may be more important consumption motivators than introduced knowledge [38]. The overarching role of women in the process of promoting traditional foods cannot be overemphasised [31, 39]. Therefore, considering the role of women in the household food choices, it is necessary to target women in order to increase the traditional food consumption patterns.

### Barriers to traditional food consumption

The strongest barriers were (1) lack of convenience or knowledge on traditional food preparation, (2) location of the residential area, and (3) aggressive advertising of processed foods. The same findings were also found in Northern Quebec in Canada where non-convenience of traditional food was among the key barriers to the consumption of traditional foods [29].

The fact that people associate indigenous, native foods with a long cooking time makes this a key barrier to the consumption of traditional dishes. Therefore, the decline in the consumption of traditional foods could in Zimbabwe be a result of the failure by the older generations to pass on the important traditional food preparation and cooking skills and the advent of extensive marketing of ultra-processed “fast foods” targeting the younger generation. Young people are likely to have never been introduced to certain traditional foods, especially those that have not made their way into the food markets. This results in a subsequent preference of younger people to fast foods as these tend to have aggressive marketing regimes.

Still, our current results are encouraging considering that cost or affordability was not cited as a key barrier in this study. This finding agrees with findings from South Africa that indeed traditional foods are regarded as “poor man’s foods” [40]. Future studies are recommended to explore the complex mechanisms underlying the decline in the consumption of traditional foods to inform policy and programming decisions. Studies have shown that the transmission of indigenous knowledge across generations is key in preserving the knowledge, attitudes, practices, and perceptions around these foods [41]. In certain countries and cultures, documentation has been astute to preserve the heritage around these foods [41–43]. We recommend the same for Zimbabwe traditional foods.

Although not significant our results showed that a relatively large fraction (34.2%) of the participants who consumed traditional foods daily were of normal weight as compared to only 15.8% who were obese. Although the frequency of traditional food consumption might be an insufficiently sensitive factor to reveal an association, we postulate that consumption of traditional foods will likely be associated with healthy eating and normal weight. For example, in the Korean culture, traditional food is normally considered as the first medicine [44]. In addition, studies conducted in South Africa [45] and Ethiopia [46] reported that food security and dietary diversification can be attained by exploiting indigenous food species. Therefore, future studies to investigate these associations are warranted.

### The theoretical contribution of the study

**Fig. 2:**
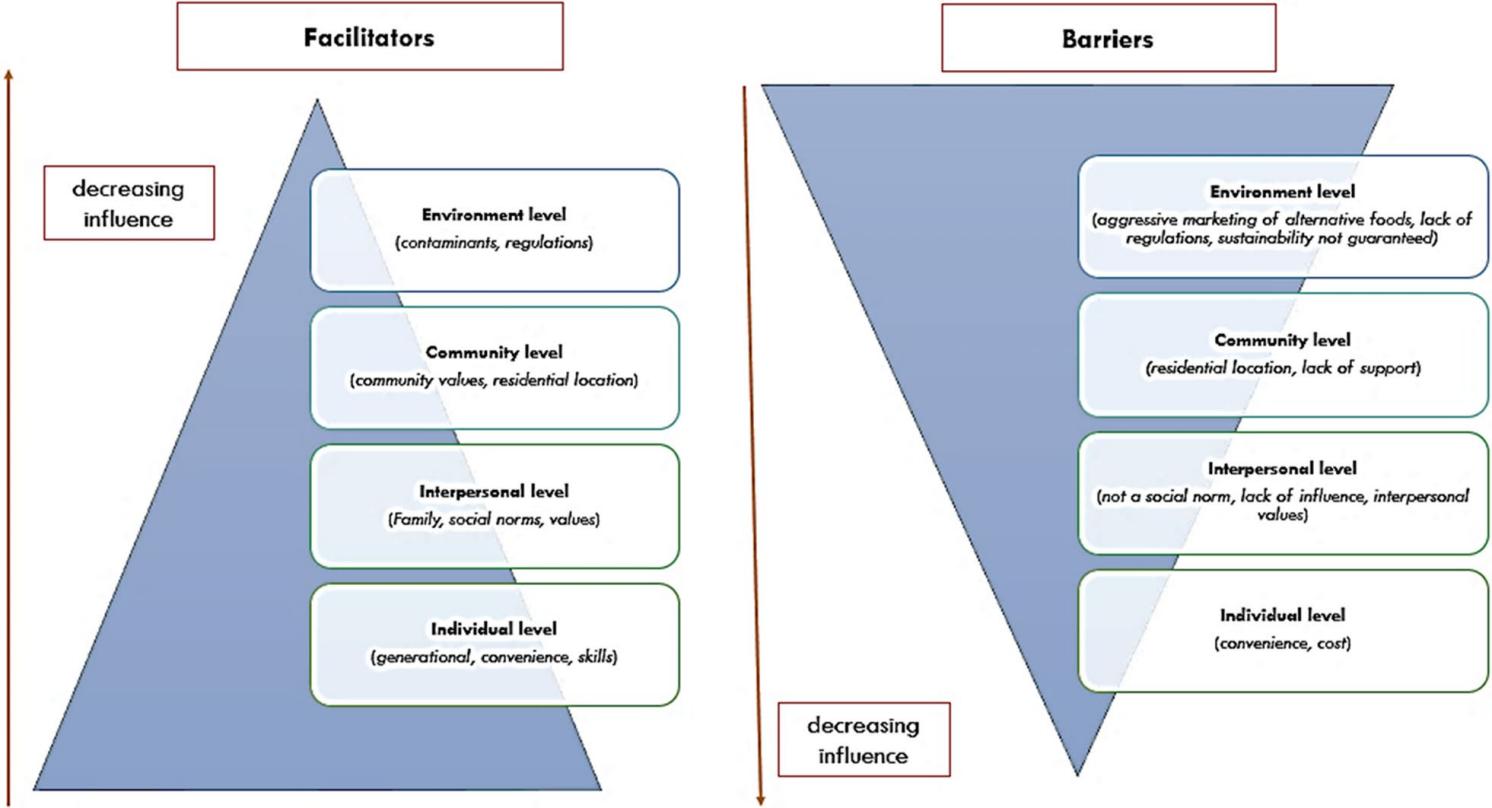
Framework for design of interventions on possible factors that influence consumption of traditional foods in Zimbabwe. *Source*: Author’s compilation

The ecological model by Gaudin et al. [29] was used as the main theoretical background for our study. It allowed most influencers and barriers to be categorised appropriately. This model provides an ideal framework that can be applied to investigate traditional food consumption especially among indigenous and minority populations [30]. In our results, the strongest facilitators came from the individual and interpersonal level (generational, family influence, convenience, and to a less extent skills) (Fig. 2). These will have to be deliberately targeted in any interventions to promote increased consumption of traditional foods in this and related settings. These findings agree with results reported by Roudsari et al. [31] in their study about barriers and facilitators to traditional food choice by Iranian women. The concepts they identified as barriers and facilitators were cultural context (customary beliefs, family teachings), social motivations (interest of family members and prioritisation), convenience, skills of food preparation, and religious considerations. Our study however did not consider the category of religion. In a study by Blesic et al. [47], the sensory appeal was a key motivation for selecting a traditional food. This was not reflected in our study. The authors also identified motivators such as convenience, price, familiarity, mood, and health concerns. These all appear to be facilitators at the individual level. Based on previous studies’ theoretical constructs, we submit that religion and sensory appeal are strong determinants that should not be ignored in setting up interventions to promote traditional food consumption. The strongest barriers in our study were identified at the community and environment levels (Fig. 2). Though we have used the same concepts from the ecological model, the ecological model makes use of concentric circles, however, here we propose a different theoretical framework (order of concepts) and strength of influence as emerging from our data analysis (Fig. 2). Interventions targeted at facilitating uptake must focus on the individual and interpersonal levels. On the contrary, to create an enabling environment to eliminate inherent barriers to consumption of traditional foods, interventions should focus on community and environmental levels. This framework can be used to direct public health nutrition efforts on the most influential level. The existing regional or provincial variations in Zimbabwe’s traditional cuisines should not be ignored in formulating sustainable interventions [48].

### Limitations of the study

The major limitation of this study is that we used a self-administered online questionnaire. The sample size of participants may not be representative of the Zimbabwean population, the results indicated a clear bias towards individuals who reside in urban areas. However, the results remain useful considering that this online survey was carried out during the peak of the COVID-19-induced lockdown where face-to-face enrolment was not possible further, the restrictive cost of mobile phone data could have attributed to the slight bias to the urban population. In addition, the pandemic and control measures made it impossible to take anthropometric measurements such as weight, height, and waist circumference. Although the BMI-SMT technique sufficed, it records what the individuals perceive to be their body size, not what exactly they weigh. It has however been recommended as a valid estimate of body perception [34].

## Conclusions

Consumption of traditional foods was low in general. The majority of participants consumed traditional foods at least once a month despite these foods still being abundant a scenario reflective of nutrition transition. Generational factors, family contribution, and food safety impact the consumption of traditional foods among adults in Zimbabwe. The time and cost required to prepare these foods largely result in them being abandoned for foreign, faster cooking foods. The food environment, particularly commercial advertising of alternative foods, is a deterrent. Misconceptions regarding traditional and indigenous foods do exist in Zimbabwe. Based on our results, the main theoretical lesson is that the strongest facilitators are at the individual and interpersonal level (generational, family influence, convenience, and to a less extent cooking skills). These will have to be deliberately targeted in any community-based nutrition education interventions to promote increased consumption of traditional foods in this and related settings.

## Data Availability

All data are available on request from the authors.
